# Preparation of an Ester-Based Polymeric Hydration Temperature Rise Inhibitor and Its Action Mechanism on Cement Hydration

**DOI:** 10.3390/polym18131603

**Published:** 2026-06-28

**Authors:** Quanwei Li, Ting Li, Xiaoning Li, Haifeng Mei, Jiaji Chen, Meixia Liu, Chaoqiang Yan

**Affiliations:** 1 CNBM Zhongyan Technology Co., Ltd., Beijing 100024, China; liquanwei@cbma.com.cn (Q.L.); lixiaon.good@163.com (X.L.); m18609636616@163.com (H.M.); p1281677823@163.com (J.C.); 17862903806@163.com (M.L.); ycq123ahi456@163.com (C.Y.); 2 China Building Materials Academy Co., Ltd., Beijing 100024, China

**Keywords:** thermal cracking, ester-based functional group, cement hydration, complexation, hydration retardation

## Abstract

To address the thermal cracking induced by concentrated heat release during early cement hydration, a novel Ester-based Polymeric Hydration Temperature Rise Inhibitor (ETRI) was designed and synthesized in this study based on the slow hydrolysis property of ester functional groups. After its incorporation into cementitious materials, the influence of ETRI on the hydration kinetics and the microstructure of hardened paste was systematically investigated. The results demonstrate that ETRI effectively regulates the heat release profile of cement hydration by prolonging the induction period. Specifically, at a dosage of 1.5%, ETRI reduces the peak heat release rate by 70.32% and the 72 h cumulative heat release by 70.82%. Mechanistic investigation reveals that ETRI undergoes slow hydrolysis in the alkaline environment to generate carboxyl groups that complex with Ca^2+^; the resulting calcium carboxylate complexes then adsorb onto the surfaces of cement particles, thereby retarding the hydration heat release process. Furthermore, with ETRI dosages not exceeding 1.5%, the promoted later-age hydration yields more hydration products, which refine the pore structure and increase the harmless pore proportion to 53.97%, thereby achieving a synergistic improvement in both the microstructure and the late-age mechanical properties of the cementitious materials. Compared with conventional retarders, ETRI exhibits a stronger inhibitory effect on the heat release of cement hydration.

## 1. Introduction

Thermal cracking induced by cement hydration heat has long been a critical technical challenge in the construction of mass concrete structures, such as hydraulic dams, bridge piers, and high-rise buildings [1,2,3]. This problem primarily arises because, during early-age hydration, the rapid reactions of mineral phases, particularly tricalcium silicate (C_3_S) and tricalcium aluminate (C_3_A), cause a pronounced internal temperature rise within the concrete. As this heat dissipates slowly, a temperature gradient develops between the interior and the exterior, which generates thermal stress and, consequently, leads to cracking, thereby compromising the structural durability of the concrete [4,5,6].

Traditional measures to mitigate thermal cracking in mass concrete include embedding cooling pipes [7], pre-cooling of aggregates [1], segmented casting [8], and surface thermal insulation [9]. However, these methods considerably increase construction complexity and cost, which limits their widespread application. Currently, another common approach is to control the internal temperature rise by using low-heat cement and supplementary cementitious materials [10,11]. Fly ash serves as a representative example: it undergoes slow hydration and releases relatively little heat; therefore, partially replacing cement with fly ash (which may reduce the water demand of the system) can lower the total heat generated by cement hydration. Nevertheless, an excessively high replacement ratio leads to problems such as insufficient early-age strength and increased susceptibility to carbonation. In addition, chemical admixtures [12,13,14,15], such as retarders, phase change materials, and expansive agents, can, to a certain extent, prolong the setting time, absorb part of the heat, and compensate for the shrinkage-induced effects of concrete. However, these measures do not fundamentally resolve the cracking problem. From the perspective of hydration kinetics, they fail to alter the heat release characteristics of early-age cement hydration. In particular, retarders merely delay the appearance of the main hydration peak without effectively reducing the peak heat flow [16]. Some researchers have proposed [17,18,19,20,21,22,23,24] that incorporating hydration heat control materials into cement can effectively reduce the main hydration heat peak and the cumulative heat release, thereby lowering the temperature gradient within the concrete and mitigating the risk of cracking. Among these materials, hydration heat inhibitors, as chemical-functional admixtures that effectively suppress the main hydration peak, enable the control of cement hydration at the source without altering the binder content, thus reducing the risk of temperature-induced cracking in concrete.

Currently, a wide variety of hydration heat inhibitors exist, yet most are starch- or dextrin-based. These inhibitors are primarily composed of modified starch or dextrin, and their molecular structures contain abundant carboxyl and hydroxyl groups. These functional groups can complex with Ca^2+^ ions generated during cement hydration, thereby reducing the Ca^2+^ concentration in the pore solution and hindering the hydration process. This type of hydration heat inhibitor offers the distinct advantages of a low dosage requirement and pronounced heat reduction. It has been reported that [20], at a dosage of 0.6%, it reduces the maximum heat evolution rate of cement hydration by 42.3% and lowers the peak temperature from 35.9 °C to 24.9 °C, without affecting the 28 d cumulative heat release or the compressive strength of concrete. From a microstructural perspective, the starch-based admixture retards the hydration of C_3_S, resulting in an 18.6% decrease in the C–S–H content. Starch-based hydration heat inhibitors also exhibit a distinctive “inhibitory effect”, which manifests as follows: in the solid state, they act as an inhibitor, reducing the heat evolution rate of the main hydration peak without significantly delaying the induction period; in the pre-dissolved state, they behave as a retarder, merely prolonging the acceleration period of hydration, similar to conventional retarders, with limited influence on the shape of the main hydration peak [17]. To improve the dispersibility of inhibitors within the cementitious system, liquid-type hydration heat inhibitors undoubtedly present a more favorable alternative. A liquid inhibitor based on modified sorbitol can regulate the hydration process by both delaying the appearance of the maximum heat evolution rate and reducing its magnitude. At dosages of 1.5% and 2.0%, the maximum heat flow was reduced by 42% and 74%, and the 48 h cumulative heat release was decreased by 63% and 84%, respectively [18]. Although the above studies employed different inhibitor forms and dosage strategies, the underlying mechanisms of their action are all intimately related to the type of functional groups in the molecular structure. It has been shown [19] that different functional groups exert distinct inhibitory effects on cement hydration. For example, compounds containing carboxyl or hydroxyl groups complex with Ca^2+^ and adsorb onto cement particle surfaces, whereas compounds containing ester groups hydrolyze in the alkaline cement paste to generate carboxyl groups. The hydrolysis rate of these ester-based compounds varies dynamically with the temperature generated by cement hydration, thereby enabling self-adaptive regulation of the hydration process. This finding demonstrates that, owing to their unique functional groups, ester-based compounds possess significant potential for temperature control in cementitious systems.

Capitalizing on the ability of ester groups to undergo controlled hydrolysis in an alkaline environment and gradually release carboxyl groups, the excessive complexation of Ca^2+^—which would otherwise lead to severe retardation—can be avoided, while effective hydration inhibition that self-attenuates at later stages is simultaneously achieved. Based on this characteristic, an ester-based polymeric hydration temperature rise inhibitor (ETRI) was designed and synthesized in the present study. The hydration behavior of cement pastes with varying ETRI dosages and the resulting properties of hardened paste were systematically investigated by means of isothermal calorimetry, X-ray diffraction analysis (XRD), thermogravimetric analysis (TGA), inductively coupled plasma optical emission spectrometry (ICP-OES), Mercury intrusion porosimetry (MIP), and scanning electron microscope (SEM). Through these investigations, the action mechanism of ETRI in cementitious materials was elucidated. This study contributes to addressing the challenge of thermal cracking in mass concrete and provides both a scientific foundation and technical support for the development of novel hydration heat control materials.

## 2. Materials and Methods

### 2.1. Raw Materials

The maleic acid (MA, AR), ethylene glycol (EG, AR), hydroquinone (HQ, AR), and p-toluenesulfonic acid (PTS, AR) used in this study were all supplied by Guangdong Wongjiang Reagent Co., Ltd. (Guangdong, China). The cement used was a reference cement, provided by China Building Materials Academy Co., Ltd. (Beijing, China), and its chemical and mineral compositions are listed in Table 1 (It should be noted that C_3_S, C_2_S, C_3_A and C_4_AF are the main mineral phases of the cement clinker). The standard sand was produced by Xiamen ISO Standard Sand Co., Ltd. (Xiamin, China).

### 2.2. Preparation and Characterization of the ETRI

#### 2.2.1. Preparation of ETRI

A certain amount of MA, EG, HQ and deionized water were added to the bottom of a four-neck round-bottom flask. After a condenser (all the glassware described above was sourced from Sichuan Shubo Group Co., Ltd., Sichuan, China) was connected, nitrogen gas was introduced to ensure that the entire experiment was conducted under a nitrogen atmosphere. The four-necked flask was heated to 65 °C, and then PTS (MA: EG: HQ: PTS: deionized water = 1.5: 1: 0.15: 0.07: 0.2) was added. The flask was further heated to 120 °C and held at this temperature for 3 h. After the reaction, the product was washed with deionized water at 60 °C and filtered three times to remove unreacted maleic acid and ethylene glycol. Subsequently, vacuum distillation was carried out at 80 °C for 0.5 h to remove excess water (ensuring the accuracy of the ETRI solution concentration), yielding pure ETRI. Finally, deionized water at 60 °C was added to dissolve the ETRI to form a 50% ETRI solution, which was then cooled and stored for subsequent direct application in cement paste systems. Table 2 shows the basic properties of ETRI.

#### 2.2.2. ^1^H NMR Characterization

**Figure 1:**
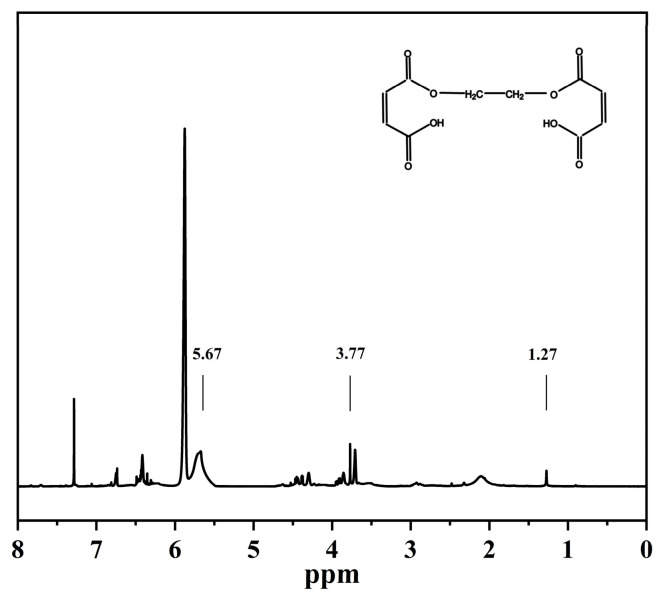
^1^H NMR spectrum of ETRI.

The ^1^H NMR spectrum of ETRI was recorded using a Bruker Plus 600 MHz nuclear magnetic resonance spectrometer (Bruker Corporation, Karlsruhe, Germany) with chloroform as the solvent, and the result is shown in Figure 1. Analysis of the spectrum reveals that the peaks at 1.27 ppm, 3.77 ppm, and 5.67 ppm correspond to the protons of –CH_2_–, –CH_2_–O–CO−, and –CH=CH−, respectively [25]. This confirms the presence of ester groups in the product.

#### 2.2.3. FTIR Characterization

**Figure 2:**
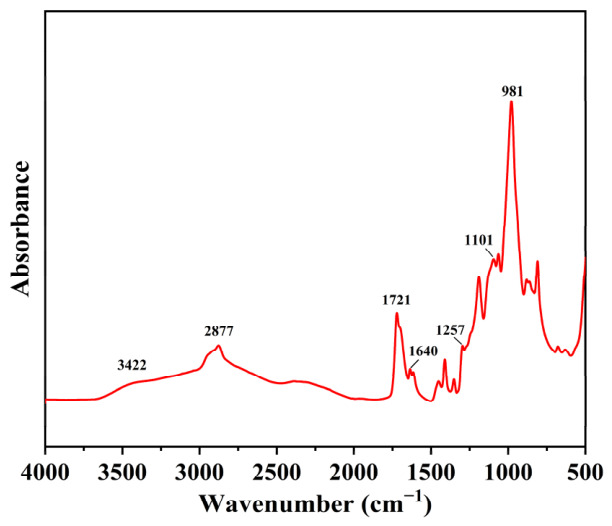
FTIR Spectrum of ETRI.

The functional groups of ETRI were analyzed using a Thermo Scientific Nicolet iS20 Fourier transform infrared spectrometer (FTIR, Thermo Fisher Scientific, Waltham, MA, USA), and the result is shown in Figure 2. The FTIR spectrum of ETRI exhibits a strong characteristic absorption peak at 1721 cm^−1^, which is assigned to the stretching vibration of the carbonyl C=O in the ester group (–COO–). The two characteristic absorption peaks at 1257 cm^−1^ and 1101 cm^−1^ correspond to the stretching vibrations of the C–O bonds in the ester group, indicating that the product contains ester groups.

#### 2.2.4. Molecular Weights and Distributions

**Figure 3:**
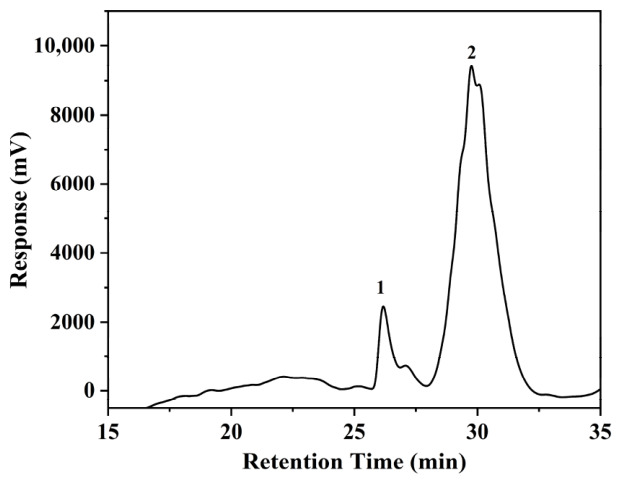
Molecular weight distribution of ETRI measured by GPC.

The molecular weight and its distribution of ETRI were determined using a Waters 2695 chromatograph (GPC, aqueous phase, Milford, MA, USA). An aqueous sodium azide solution was used as the eluent, with an injection volume of 100 μL and a flow rate of 1 mL/min. As shown in Figure 3 and Table 3, ETRI exhibits two molecular weight distributions. The main distribution, Peak 2, has a weight average molecular weight (Mw) of 294 g/mol, accounts for 82.23% of the total, and possesses a polydispersity index (PDI) of 1.02, indicating an extremely narrow molecular weight distribution and good uniformity. Peak 1 likely corresponds to ester oligomers.

Overall, combined with the FTIR and ^1^H NMR analysis results, the esterification reaction of ETRI proceeded as designed.

### 2.3. Sample Preparation and Test Methods

#### 2.3.1. Setting Times

In accordance with the Chinese national standard GB/T 1346-2024, Test methods for water requirement of normal consistency, setting time and soundness of the Portland cement [26], the setting time of cement pastes with different ETRI dosages (effective solid content of ETRI ranging from 0% to 2% by weight of cement, with the dosage calculated based on solid content and the water introduced by the ETRI solution deducted accordingly) was determined using a Vicat apparatus. The initial setting time was defined as the time required for the needle to penetrate to a depth of 4 mm ± 1 mm from the base plate, and the final setting time was defined as the time required for the needle to penetrate only 0.5 mm into the paste and for the annular attachment to cease leaving an impression on the specimen surface. Three specimens were prepared for each mix proportion, and the test results were averaged.

#### 2.3.2. Mechanical Strength

Mortar specimens with different ETRI dosages were prepared and cured in accordance with the method specified in GB/T 17671-2021, Test method of cement mortar strength (ISO method) [27]. After curing to different ages, the compressive strength and flexural strength were tested. Three specimens were prepared for each mix proportion, and the test results were averaged.

#### 2.3.3. Cement Hydration Heat Test

The hydration heat of cement pastes with different ETRI dosages was measured using a TAM III eight-channel microcalorimeter (TA Instruments, New Castle, DE, USA). For each measurement, 4 g of cement was weighed into a sample ampoule, and an ETRI solution (ETRI at 0–2% by mass of cement) was added at a water-to-cement ratio of 0.4. The measurement was initiated simultaneously with stirring, and the heat release was recorded. The initial test temperature was 25 °C.

#### 2.3.4. XRD Characterization

Samples with different ETRI dosages and ages were tested using a Bruker D8 Focus X-ray diffractometer (Bruker Corporation, Karlsruhe, Germany). Quantitative analysis was performed using TOPAS V5 software. The samples with different ETRI dosages and ages were first hydration-stopped with isopropanol, then dried and ground. Rutile was used as an internal standard. After passing through a 200-mesh sieve, the samples were tested. The sample test channel employed a copper target, operated at a voltage of 40 kV and a current of 100 mA, with a scanning angle range of 5–90° and a scanning speed of 2°/min.

#### 2.3.5. Thermal Behavior

Thermal behavior analysis of samples with different ETRI dosages and ages was performed using a Netzsch TG 209 thermogravimetric analyzer (NETZSCH-Gerätebau GmbH, Selb, Germany). The sample preparation method was the same as that for XRD. During the test, the heating rate was 10 °C/min, the temperature range was 30–1000 °C, and the atmosphere was nitrogen. The mass fraction of CH was calculated according to Equation (1), taking into account the carbonation of CH:

(1) $$M_{CH}=L_{CH}\times \frac{74}{18}+L_{CC}\times \frac{74}{44}$$

where *M_CH_* is the total mass fraction of CH (%), *L*_CH_ is the mass loss of CH (%), and *L*_CC_ is the mass loss of CaCO_3_ (%).

#### 2.3.6. Inductively Coupled Plasma Optical Emission Spectrometry

The changes in the concentrations of Ca, S, Si, and Al elements in the cement pore solution were analyzed by inductively coupled plasma optical emission spectrometry (ICP-OES, SPECTROBLUE, SPECTRO Analytical Instruments, Kleve Germany). Cement was mixed separately with deionized water and with an ETRI aqueous solution (ETRI at 1.5% by mass of cement) to form pastes, using a water-to-cement ratio of 0.5, and then sealed for storage. At specific ages (0.5 h, 1 h, 1.5 h, 2 h, 2.5 h, and 3 h), a portion of the paste was taken out and centrifuged at 5000 r/min; the supernatant was then collected for elemental concentration determination.

#### 2.3.7. Mercury Intrusion Porosimetry Test

The pore structure of hardened cement paste samples with different ETRI dosages at 28 days was analyzed using a Micromeritics AutoPore V 9620 high-performance automatic mercury intrusion porosimeter (MIP, Micromeritics Instrument Corporation, Norcross, GA, USA).

#### 2.3.8. Scanning Electron Microscopy Testing

The micro-morphology of samples with different ETRI dosages and ages was observed using a Quanta 250 FEG environmental scanning electron microscope (SEM, FEI Company, Hillsboro, OR, USA). The accelerating voltage was 15.0 kV, and the resolution was 2.0 nm. The sample preparation and hydration stopping procedures were the same as those for XRD. After hydration stopping, the samples were broken into small pieces using a tool, and the morphology of the fractured surfaces was observed.

## 3. Results and Discussion

### 3.1. Setting Time and Mechanical Strength

Table 4 shows the water requirement for normal consistency and the setting time of cement pastes with different ETRI dosages. As can be seen from the table, the water requirement for normal consistency decreased progressively with increasing ETRI dosage, indicating that ETRI possesses a certain dispersing and water-reducing effect. Compared with the control group, at a low ETRI dosage (≤1.0%), the initial and final setting times were prolonged by 12–21 min and 20–41 min, respectively, exhibiting a retarding behavior. In contrast, when the ETRI dosage exceeded 1.5%, the initial and final setting times gradually shortened, becoming lower than those of the control. This phenomenon can likely be attributed to the fact that the pastes containing ETRI generated a larger amount of heat within a short period (see Section 3.2), which induced false setting and thus manifested macroscopically as a reduction in setting time. Such false setting also leads to a slow strength development in the specimens.

**Figure 4:**
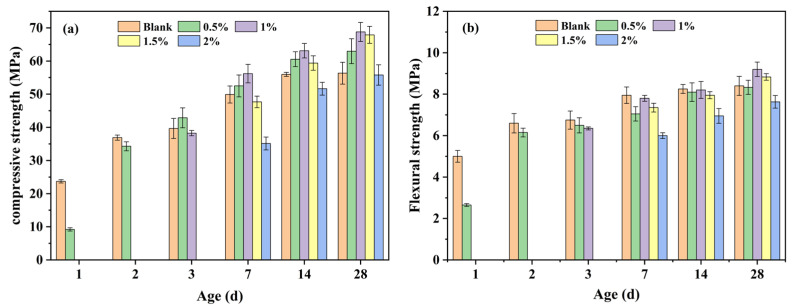
Effect of ETRI content and age on the (**a**) compressive strength and (**b**) flexural strength of mortar.

Figure 4 shows the evolution of compressive strength and flexural strength of mortars with different ETRI dosages at various curing ages. As the curing age increased, the compressive and flexural strengths of all mortar groups exhibited an increasing trend; however, the incorporation of ETRI significantly influenced the early-age strength development. In the early curing stage before 3 d, the strength development of the mortar was relatively slow. Specifically, when the ETRI dosage exceeded 1.0%, the specimens could not even be demolded, exhibiting no measurable strength. At the curing age of 28 d, the compressive strengths of mortars with ETRI dosages of 0.5%, 1.0%, and 1.5% increased by 11.74%, 22.03%, and 20.50%, respectively, compared with the control group, while the flexural strengths changed by −0.79%, 9.52%, and 5.16%, respectively. However, When the dosage was increased to 2.0%, the compressive strength remained essentially unchanged, whereas the flexural strength decreased by 9.13%. These results indicate that, within an appropriate dosage range (≤1.5%), ETRI exerts a strong inhibitory effect on cement hydration at the early stage; however, as the curing age increases, this negative effect gradually diminishes and can be transformed into strength enhancement at later ages.

### 3.2. Analysis of Cement Hydration Heat Evolution

**Figure 5:**
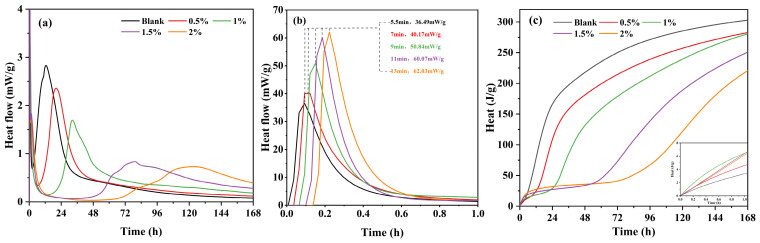
Hydration heat of cement with different ETRI dosages: (**a**) heat flow rate; (**b**) heat flow rate from 0 to 1 h; (**c**) cumulative heat release.

Figure 5 shows the heat evolution rate and cumulative heat release curves of cement hydration with different ETRI dosages.

From Figure 5a, it can be observed that, compared with the control group, the incorporation of ETRI altered the hydration heat release behavior of cement: the appearance time of the main heat evolution peak was delayed with increasing dosage, and the peak heat evolution rate gradually decreased. Specifically (as shown in Table 5), upon the addition of 0.5% and 1.0% ETRI, the appearance times of the main peak were prolonged to 20.15 h and 32.33 h, respectively, and the peak heat evolution rates were reduced by 16.61% and 39.93%, respectively, compared with the control group (2.83 mW/g). When the dosage was increased to 1.5% and 2.0%, the appearance times were further postponed to 79.38 h and 122.48 h, and the peak heat evolution rates decreased by 70.32% and 74.20%, respectively. These observations indicate that this hydration heat behavior exerts a certain influence on the early hydration process, inhibiting the formation of hydration products, which is consistent with the mortar strength development. This phenomenon can be attributed to the complexation between Ca^2+^ in the cement pore solution and the active functional groups (such as carboxyl and hydroxyl groups) generated by the hydrolysis of ETRI in the alkaline environment. While reducing the supersaturation of Ca^2+^ in the liquid phase, the resulting carboxylate–calcium complexes adsorb onto the surfaces of unhydrated particles and early hydration products, forming a physical barrier that further hinders cement hydration, thereby lowering the hydration heat evolution rate and the early cumulative heat release [19,28].

Figure 5c shows that, in the very early stage of hydration (0–1 h), the cumulative heat release of the pastes incorporating ETRI was consistently higher than that of the control group and increased with increasing dosage. This phenomenon can be attributed to the “dissolution-promoting” effect of ETRI: ETRI molecules and their early hydrolysis products (small amounts of –COOH and –OH) enhance the dispersibility and dissolution contact area among cement particles through electrostatic adsorption and steric hindrance [29], while simultaneously disrupting the local dissolution equilibrium of minerals through weak complexation with Ca^2+^, thereby promoting the initial dissolution of minerals such as C_3_A and C_3_S. Consequently, more heat is released within a very short time, which also explains why the first heat evolution peak of the cement paste increases with higher ETRI dosage (see Figure 5b) [30]. At 72 h of hydration, the cumulative heat releases of pastes with ETRI dosages of 0.5%, 1.0%, 1.5%, and 2.0% were reduced by 14.18%, 28.48%, 70.82%, and 83.66%, respectively, compared with the control group. It is noteworthy that, as hydration proceeds, the cumulative heat release of the cement pastes incorporating ETRI gradually approaches that of the control group, indicating that the inhibitory effect of ETRI on later-age hydration gradually weakens and that the hydration reactions can continue after the delay. That is, the reduction in cumulative heat release is partly attributable to the retardation of the hydration reactions, rather than to permanent inhibition. This can be explained by two aspects: on the one hand, the ester groups in ETRI molecules undergo continuous hydrolysis in the alkaline environment; once the ester groups are consumed through hydrolysis, no new –COOH groups are generated to complex with Ca^2+^, and the inhibitory activity gradually diminishes. On the other hand, the adsorbed film previously formed on the surfaces of cement particles is gradually encapsulated or covered by hydration products, or undergoes self-degradation, progressively losing its ability to inhibit hydration, thereby allowing later-age hydration to recover [19,31]. This “self-attenuation” characteristic is the essential difference between ETRI and conventional retarders: ETRI can effectively regulate the concentrated heat release of early cement hydration without compromising the later-age hydration degree and mechanical properties.

### 3.3. Phase Analysis of Hydration Products

#### 3.3.1. XRD Analysis

**Figure 6:**
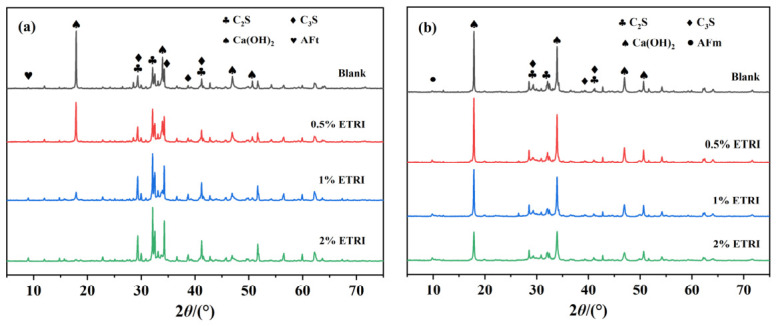
X-ray diffraction patterns of hardened cement paste with different ETRI dosages: (**a**) 24 h; (**b**) 28 d.

Figure 6 and Table 6 present the X-ray diffraction patterns and quantitative phase analysis results of hardened cement pastes with different ETRI dosages at 24 h and 28 d. It can be seen that the incorporation of ETRI did not alter the types of hydration products.

From Figure 6a and Table 6, at the age of 24 h, compared with the control cement, the incorporation of ETRI led to increases, to varying degrees, in the contents of unhydrated C_3_S and C_3_A, while the content of CH decreased significantly. At an ETRI dosage of 2.0%, the CH content decreased from 20.12% in the control to 2.96%, a reduction of 85.30%. This indicates that at an early age (1 d), ETRI retards the cement hydration process mainly by inhibiting the hydration of C_3_S and C_3_A, and the degree of retardation increases with ETRI dosage. This phase evolution pattern is highly consistent with the observation from hydration heat analysis that ETRI prolongs the induction period, postpones the main heat evolution peak, and reduces early cumulative heat release, and thus further confirms the inhibitory effect of ETRI on early cement hydration.

At the age of 28 d (Figure 6b), the degree of hydration of all samples increased markedly, with a notable rise in the CH content. It is noteworthy that the CH content in the pastes incorporating ETRI was higher than that of the control (45.54%); specifically, the 1% dosage group reached 50.14%, representing a 42.40% increase over the control. However, when the ETRI dosage reached 2.0%, the degree of hydration declined, and the CH content decreased by 21.93% compared with the control group, which is consistent with the strength development pattern at 28 d. This phenomenon further reveals the action mechanism of ETRI on cement hydration: at an early age, ETRI primarily decreases the hydration heat peak and the heat release by inhibiting the hydration of C_3_A and C_3_S; as the curing age increases, the inhibitory effect on cement hydration gradually diminishes due to the “self-attenuation” [31] characteristic of ETRI and the loss of inhibitory activity of its hydrolysis products, and the cement particles whose hydration was delayed at an early stage continue to hydrate in the subsequent period, generating more hydration products and thereby laying the foundation for later-age strength development. It should be noted that an excessive amount of ETRI will over-suppress cement hydration and negatively affect the strength.

#### 3.3.2. TG-DTG Analysis

**Figure 7:**
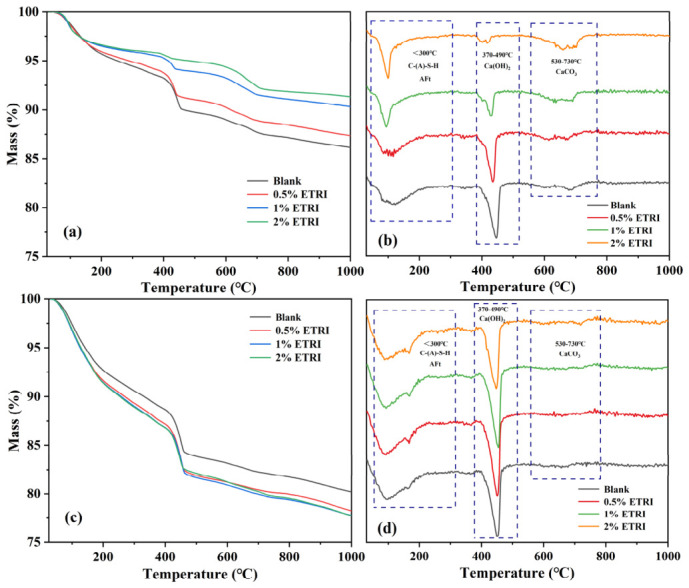
TG-DTG curves of hardened cement paste with different ETRI dosages: (**a**) TG at 24 h; (**b**) DTG at 24 h; (**c**) TG at 28 d; (**d**) DTG at 28 d. (The blue dashed box indicates the temperature range for the TG analysis).

Figure 7 and Table 7 present the TG-DTG curves and the mass losses in each temperature range of hardened cement pastes with different ETRI dosages at 24 h and 28 d. The mass loss of the samples can be divided into three main stages: the 0–300 °C range corresponds to the dehydration of gel phases such as calcium (alumino)silicate hydrate (C-(A)-S-H) and ettringite (AFt); the 370–490 °C range corresponds to the decomposition of CH; and the 530–730 °C range corresponds to the decomposition of CaCO_3_ (formed by the carbonation of CH [19,32].

In cement paste hydration, the CH content reflects the degree of hydration and can be calculated using Equation (1) (see Table 7). As shown in the table, at the early age of 24 h, the mass loss of CH gradually decreased with increasing ETRI dosage. At dosages of 0.5%, 1.0%, and 2.0%, the CH mass loss was reduced by 14.91%, 40.01%, and 57.14%, respectively, compared with the control. Meanwhile, the mass loss of gel phases such as C-(A)-S-H and AFt also decreased with increasing ETRI dosage. This result further corroborates the XRD analysis and indicates that ETRI inhibited the early hydration reactions, with the degree of inhibition increasing with dosage. As hydration proceeded, the CH content increased significantly at 28 d. Compared with the control group, the samples incorporating ETRI showed a higher mass loss of CH. This phenomenon can be explained as follows: by hindering early cement hydration, ETRI provides a more sufficient time window for the dissolution and hydration of cement particles, thereby preventing the rapid and disordered precipitation of hydration products in a confined space. Consequently, the later-stage C-(A)-S-H gel is able to nucleate and grow in a more homogeneous liquid-phase environment, achieving a higher degree of hydration [33]. This trend is consistent with the strength development characteristics and the XRD phase evolution results, and together they reveal the hydration regulation mechanism of ETRI at appropriate dosages: “early inhibition, later-age hydration recovery and enhancement.”

### 3.4. Analysis of Ion Concentrations in Pore Solution

**Figure 8:**
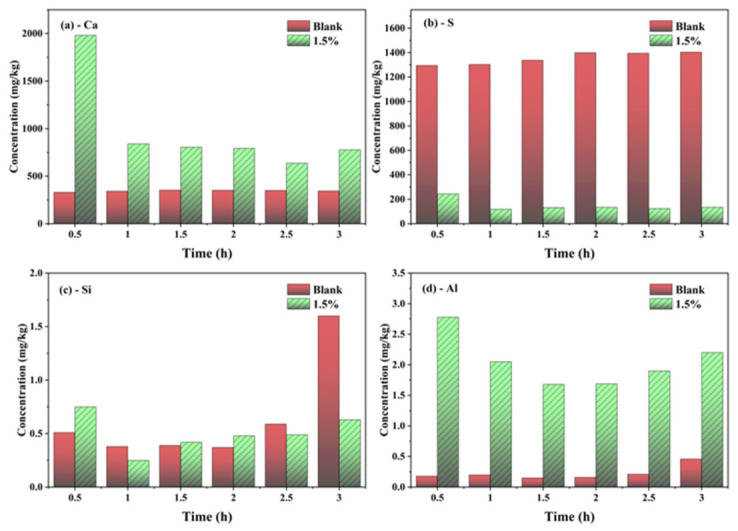
Ion concentrations in pore solution of cement paste with and without ETRI: (**a**) Ca; (**b**) S; (**c**) Si; (**d**) Al.

Figure 8 illustrates the evolution of the concentrations of major elements (Ca, S, Si, Al) in the pore solution of the control cement paste and that incorporating 1.5% ETRI during early hydration (0.5–3 h).

**Figure 9:**
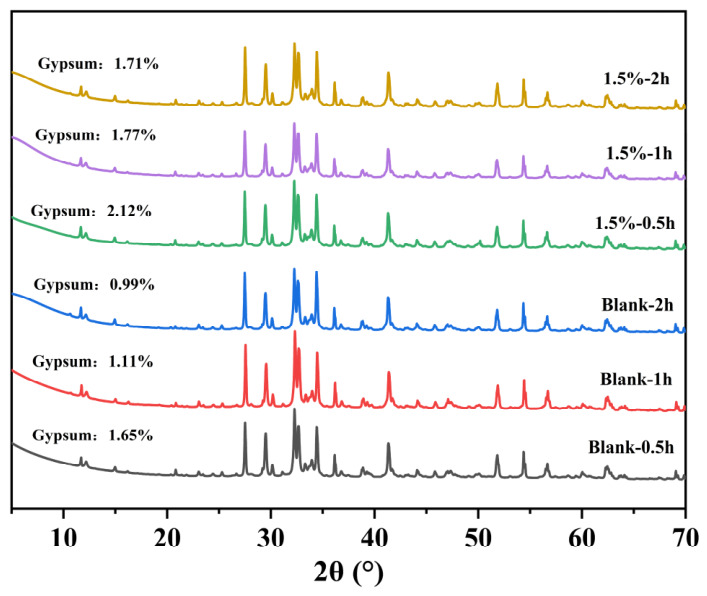
X-ray diffraction patterns and quantitative analysis of hardened cement paste with and without ETRI.

From the figure, it can be seen that during early cement hydration (0.5–3 h), the concentrations of Ca, S, Si, and Al exhibited the most pronounced changes; thus, these concentrations can, to a certain extent, reflect the influence mechanism of ETRI on the early stage of cement hydration. In the control group, the concentrations of Ca and S remained essentially at the same level throughout the test period, indicating that a dynamic equilibrium between the dissolution and precipitation of minerals (C_3_S, C_3_A, C_2_S) and gypsum was reached during early cement hydration. After the incorporation of ETRI, the Ca concentration in the pore solution reached as high as 1979.61 mg/kg at 0.5 h, approximately six times that of the control, and then decreased rapidly and stabilized. The S concentration was reduced to approximately one-fifth of that in the control, and the Al concentration was also significantly higher (by several times) than in the control. The above phenomena can be attributed to the dissolution-promoting effect of ETRI: ETRI molecules adsorb onto the surfaces of cement particles, enhancing the dispersibility among cement particles and accelerating the early dissolution of minerals. This leads to a large amount of Ca^2+^ and Al(OH)_4_^−^ entering the pore solution. Subsequently, under alkaline conditions, the polar functional groups (–COOH) released by the hydrolysis of ETRI form soluble complexes with Ca^2+^, initiating the inhibition of the cement hydration process [34,35,36]. However, due to the extremely high total Ca^2+^ concentration in the liquid phase, the free Ca^2+^ is in a supersaturated state, which in turn inhibits the dissolution-precipitation of gypsum, manifesting as a substantial decrease in the S concentration. Quantitative XRD analysis of the hardened cement paste (Figure 9) showed that the gypsum content in the paste with ETRI was consistently higher than that in the control, further supporting the above explanation.

### 3.5. Microstructure

#### 3.5.1. Pore Structure Analysis

**Figure 10:**
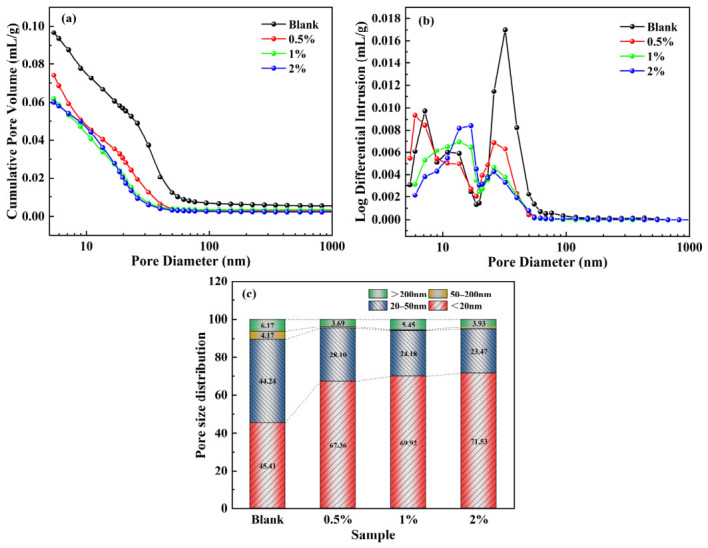
Pore structure of hardened cement paste with different ETRI dosages at 28 d: (**a**) cumulative pore volume; (**b**) pore size distribution; (**c**) statistics of pore size distribution.

The pore structure of hardened cement paste is a critical factor influencing its mechanical properties and durability. According to the effect of pore size on the performance of cementitious materials, pores can generally be classified into four categories: harmless pores (<20 nm), less harmful pores (20–50 nm), harmful pores (50–200 nm), and more harmful pores (>200 nm) [36,37,38,39,40]. The lower the porosity and the higher the proportion of harmless pores, the denser the microstructure of the hardened cement paste and the correspondingly higher its strength. Figure 10 shows the cumulative pore volume distribution, the log-differential intrusion curves, and the pore volume fractions in different pore size ranges of hardened cement pastes with different ETRI dosages.

From the cumulative pore volume distribution in Figure 10a and the log-differential intrusion curves in Figure 10b, it can be seen that the incorporation of ETRI altered the pore structure characteristics of the cement paste. As the ETRI dosage increased, the total porosity exhibited a decreasing trend. Specifically, at an ETRI dosage of 1.0%, the total porosity reached its minimum value of 11.19%, which was 36.24% lower than that of the control group (17.55%). Meanwhile, the proportion of pores larger than 20 nm (i.e., the sum of less harmful, harmful, and more harmful pores) decreased from 54.59% in the control group to 32.64%, 30.08%, and 28.47% at dosages of 0.5%, 1.0%, and 2.0%, respectively, representing reductions of 40.21%, 44.90%, and 47.85%. This indicates that the incorporation of ETRI effectively reduced the total porosity and significantly optimized the pore structure, shifting the pore size distribution toward smaller sizes (harmless pores), which is also consistent with the mortar strength development.

Overall, the optimizing effect of ETRI on the pore structure can be attributed to its regulation mechanism of hydration products: during early cement hydration, ETRI retards the hydration process through hydrolysis, adsorption, and complexation, thereby preventing the rapid accumulation of hydration products that would lead to the formation of a harmful pore structure. As the inhibitory effect of ETRI attenuates, the hydration products uniformly fill the pore space, optimizing the pore structure distribution and reducing the generation of large pores [37]. However, when the ETRI dosage is excessively high (2.0%), although the pore structure is relatively dense, the excessive complexation between the -COO^−^ groups generated by hydrolysis and Ca^2+^, as well as the surface adsorption, lead to an excessive delay of the hydration reactions, and some cement particles are not sufficiently hydrated. As evidenced by XRD, the quantity of hydration products formed at the same age is relatively low, ultimately resulting in a decrease in strength. It should be noted that, although the specimen with 2.0% ETRI exhibited the lowest total porosity, its strength did not reach the maximum value, which contradicts the classical strength–porosity relationship. The influence of pore structure may be governed by the critical pore size rather than solely by the total pore volume.

#### 3.5.2. Micro-Morphology Analysis

**Figure 11:**
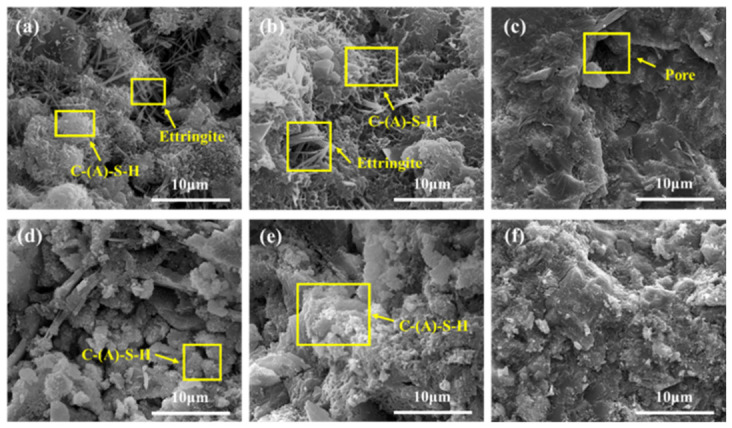
SEM images of hardened cement paste with different ETRI dosages: (**a**) Blank at 1 d; (**b**) Blank at 7 d; (**c**) Blank at 28 d; (**d**) 1% at 1 d; (**e**) 1% at 7 d; (**f**) 1% at 28 d.

Figure 11 shows SEM images of hardened cement pastes with different ETRI dosages at the ages of 1 d, 7 d, and 28 d. From Figure 11a,d, it can be seen that at the age of 1 d, the hydration products in the control sample had already covered the surfaces of cement particles, and a large number of needle-like ettringite (AFt) and C-(A)-S-H gel could be observed. In contrast, in the sample incorporating 1% ETRI, the needle-like ettringite was difficult to observe, the C-(A)-S-H gel was markedly reduced, and the microstructure was looser, which further explains the lower early-age strength of the mortar with ETRI. As the hydration age increased, the hydration products in both groups became progressively more abundant; the C-(A)-S-H gel enveloped the cement particles and bonded them into a continuous network structure, and the densification of the paste improved significantly [17]. Notably, at 28 d, the morphology of the hydration products in the sample with 1% ETRI showed no distinct difference from that of the control group. However, due to the moderate retardation of the early hydration process, the later-age C-(A)-S-H gel was filled more uniformly, reducing the overall pore structure and providing a structural basis for its strength development.

### 3.6. Analysis of Action Mechanism

**Figure 12:**
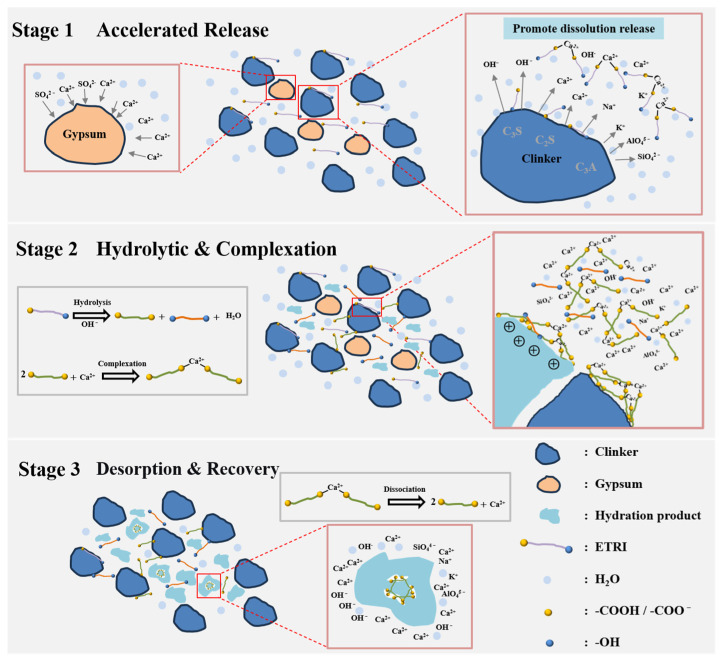
Action mechanism of ETRI in cement.

Based on the above experiments, the action mechanism of ETRI in cementitious materials is further elucidated, as schematically illustrated in Figure 12.

The regulation of cement hydration by ETRI follows a synergistic three-stage process: “accelerated release—hydrolysis complexation—dissociation recovery.”

Stage I (Accelerated release): ETRI itself is a low-molecular-weight ester-based polymer with terminal –COOH groups. During the initial dissolution of cement, ETRI molecules and their early hydrolysis products (small amounts of –COOH and –OH) improve the dispersibility of cement particles through electrostatic adsorption and steric hindrance, thereby exposing more mineral surfaces. Meanwhile, through weak complexation with Ca^2+^, they disrupt the local dissolution equilibrium of minerals, promoting the continuous dissolution of ions such as Ca^2+^ and Al(OH)_4_^−^, thus building up a high-concentration ion reservoir for subsequent hydration [38]. At this stage, owing to the high Ca^2+^ concentration in the liquid phase, the dissolution of gypsum is inhibited and the very early hydration process is retarded, which has also been confirmed experimentally. It should be noted that, although the Ca^2+^ concentration rises sharply during this stage, the subsequent rapid complexation with –COO^−^ groups (generated in Stage II) forms carboxylate–calcium complexes, which prevent the uncontrolled precipitation of coarse CH crystals caused by local supersaturation, thereby avoiding damage to the matrix structure [39,40].

Stage II (Hydrolysis complexation): In the strongly alkaline environment of the cement paste, ETRI undergoes slow and sustained hydrolysis, releasing free –COOH groups that form soluble complexes with Ca^2+^ in the solution. These complexes preferentially adsorb onto the surfaces of unhydrated particles and early hydration products (such as C-(A)-S-H and ettringite nuclei), occupying nucleation sites and thereby inhibiting the hydration process [41,42]. Consequently, the appearance of the main heat evolution peak is postponed, the heat evolution rate is reduced, and the early cumulative heat release is substantially decreased.

Stage III (Dissociation recovery): As hydration proceeds, the chemical environment of the cement liquid phase changes, leading to the dissociation of the carboxylate-Ca complexes and the release of Ca^2+^ and –COOH. At this point, although –COOH remains weakly adsorbed on the surfaces of cement particles and hydration products, it loses the strong occupancy of nucleation sites, and the hydration inhibition effect is lifted. Subsequently, newly formed hydration products (C-(A)-S-H gel, CH crystals) nucleate and grow on the surfaces bearing the residual organic layer, physically burying –COOH within the hydration products. The buried organic molecules lose their interfacial activity and no longer affect the subsequent reactions [30,43]. Then, the high-concentration ions accumulated during Stage I uniformly nucleate at new hydration active sites, generating dense C-(A)-S-H gel that fills the pores, thereby effectively controlling the risk of early-age thermal cracking without compromising later-age strength and durability.

### 3.7. Performance Comparison of ETRI and Set Retarder

Table 8 compares the effects of ETRI and three conventional retarders (sucrose, sodium gluconate, and citric acid) on the hydration heat and late-age strength of cement pastes, with data taken from the existing literature.

As shown in the table, compared with the blank sample, the addition of 1.50% ETRI resulted in a 74.12% reduction in the peak heat evolution rate and a 16.80% increase in the 28 d compressive strength. In contrast, the three retarders (each at a dosage of 0.07%) only reduced the peak heat evolution rate by 21.87–28.94% and increased the 28-day compressive strength by 8.85%. These differences indicate that, compared with conventional retarders, ETRI exerts a stronger inhibiting effect on the exothermic peak during the acceleration period of cement hydration. Moreover, this inhibition does not compromise the late-age mechanical properties but instead leads to a higher strength gain. Such a distinction makes ETRI more advantageous than conventional retarders in the temperature control and crack prevention of mass concrete.

## 4. Conclusions

In this study, an ester-based polymeric hydration temperature rise inhibitor (ETRI) was synthesized based on ester functional groups, and its influence on the mechanical properties of cementitious materials and its action mechanism on cement hydration were systematically investigated. The main conclusions are as follows:

(1). A novel Ester-based Polymeric Hydration Temperature Rise Inhibitor (ETRI) was designed and synthesized via esterification, and its molecular structure was confirmed by ^1^H NMR, FTIR, and GPC analyses to be consistent with the expected design.
(2). The incorporation of ETRI alters the setting time of the cement paste, and an appropriate dosage of ETRI significantly enhances the later-age strength of the mortar. ETRI exhibits a certain dispersing and water-reducing effect. At low dosages (≤1.0%), it acts as a retarder, whereas when the dosage exceeds 1.5%, it induces false setting of the paste. Owing to the inhibitory effect of ETRI on early-stage hydration, the early-age strength development (3 d) of the mortar was negatively affected; however, as the curing age increased, this negative effect gradually diminished and was eventually transformed into a strength enhancement at later ages. At 28 d, the compressive strength of the mortar with an appropriate ETRI dosage (1.0%) was 22.03% higher than that of the control group.
(3). ETRI altered the hydration heat release behavior of cement. Compared with the control group, the cement pastes incorporating ETRI exhibited a prolonged induction period, effectively reduced hydration heat evolution rate, and decreased early cumulative heat release (at 1.5% ETRI, the heat evolution rate was reduced by 70.32% and the 72 h cumulative heat release by 70.82%). However, an excessively high dosage (≥2.0%) over-retarded the hydration process (the main heat evolution peak was postponed by 109.92 h).
(4). ETRI reduces the main hydration heat peak and the heat release mainly by inhibiting the hydration of C_3_S and C_3_A through slow hydrolysis, complexation with Ca^2+^, and surface adsorption. For samples with an appropriate ETRI dosage, as the curing age increased, the hydration inhibition gradually weakened without hindering the formation of later-age hydration products. In contrast, an excessive ETRI dosage (2.0%) over-suppressed cement hydration, as indicated by a 21.93% decrease in CH content compared with the control at 28 d, leading to an insufficient quantity of hydration products.
(5). The incorporation of ETRI optimized the pore structure of the hardened cement paste. As hydration proceeded, an appropriate dosage of ETRI significantly optimized the distribution of hydration products, formed a denser pore structure, increased the proportion of harmless pores in the hardened cement paste at later ages (the harmless pore proportions reached 48.34%, 53.97%, and 57.52% at dosages of 0.5%, 1.0%, and 2.0%, respectively), and improved the later-age microstructure and mechanical properties of the hardened cement paste.
(6). ETRI exerts a stronger inhibitory effect on cement hydration than conventional retarders. Specifically, compared with the control, the reduction in peak heat evolution rate achieved by ETRI (74.12%) far exceeds those of three conventional retarders (21.87–28.94%), and the 28-day compressive strength is also enhanced by 16.80%.

## Figures and Tables

**Table 1:** Chemical and mineral compositions of the reference cement.

Chemical Compositions	SiO_2_	Al_2_O_3_	Fe_2_O_3_	CaO	MgO	SO_3_	Na_2_O	f-CaO	Loss on Ignition
Wt. (%)	20.54	4.78	3.38	62.58	3.60	1.98	0.59	0.70	1.83
**Mineralogical Composition**	**C_3_S**	**C_2_S**	**C_3_A**	**C_4_AF**	**f-CaO**				
Wt. (%)	58.86	15.95	6.80	11.45	0.89				

**Table 2:** Basic properties of ETRI.

Admixture	Solubility at 25 °C	Solubility at 60 °C	Storage Stability of Pure ETRI at 25 °C	Storage Stability of 50% ETRI Solution at 25 °C
ETRI	low	Good	Good	≥60 days

**Table 3:** Molecular properties of ETRI.

Peak No	Weight Average Molecular Weight (g/mol)	Number Average Molecular Weight (g/mol)	PDI *	Area (%)
1	901	871	1.03	17.77
2	294	282	1.02	82.23

* PDI (Polydispersity Index) = Mw/Mn, Mw stands for mean Weight average molecular weight, Mn stands for mean Number average molecular weight.

**Table 4:** Setting time of cement paste with various ETRI contents.

Dosage	Normal Consistency Water Demand (g)	Initial Setting Time (min)	Final Setting Time (min)
0%	27.55	167	205
0.5%	27.20	188	246
1.0%	27.10	179	225
1.5%	26.05	156	189
2.0%	26.05	122	143

**Table 5:** Hydration heat values of cement with different ETRI dosages.

Dosage	Heat Flow			Heat					
	Peak Time of Heat Release Rate (h)	Peak Heat Release Rate (mW/g)	Reduction Rate (%)	24 h		72 h		168 h	
				Cumulative Heat Release (J/g)	Reduction Rate (%)	Cumulative Heat Release (J/g)	Reduction Rate (%)	Cumulative Heat Release (J/g)	Reduction Rate (%)
Blank	12.56	2.83	/	166.06	/	250.03	/	302.85	/
0.5%	20.15	2.36	16.61	108.53	34.65	214.58	14.18	282.73	6.65
1.0%	32.33	1.70	39.93	29.04	82.51	178.82	28.48	280.76	7.30
1.5%	79.38	0.84	70.32	27.23	83.60	72.96	70.82	250.78	17.19
2.0%	122.48	0.73	74.20	31.90	80.79	40.85	83.66	220.71	27.12

**Table 6:** Quantitative phase composition from XRD analysis of hardened cement paste with different ETRI dosages (wt.%).

Age	Dosage	C_2_S	C_3_S	C_4_AF	C_3_A	CH	AFt	AFm
1 d	Blank	18.54	35.26	14.25	1.22	20.12	1.22	/
	0.5%	14.52	47.88	10.92	6.86	14.74	2.08	/
	1.0%	10.57	56.81	10.08	6.44	8.50	4.32	/
	2.0%	10.57	64.15	8.58	5.69	2.96	4.97	/
28 d	Blank	18.97	28.08	10.54	2.88	45.10	/	3.56
	0.5%	16.96	17.68	5.52	0.77	45.54	/	5.16
	1.0%	18.21	11.15	5.98	0.03	50.14	/	5.92
	2.0%	22.64	12.23	5.49	2.01	35.21	/	5.66

**Table 7:** Mass loss of CH in hardened cement paste with different ETRI dosages (wt.%).

	24 h				28 d			
Dosage	Blank	0.5%	1.0%	2.0%	Blank	0.5%	1.0%	2.0%
0 °C–300 °C	5.55	5.10	4.09	3.93	9.40	10.68	10.99	11.10
370 °C–490 °C	3.77	3.08	1.62	0.86	5.24	5.79	5.67	5.18
530 °C–730 °C	2.15	2.14	2.40	2.77	1.56	1.46	1.75	2.01
Total mass of CH	19.11	16.26	10.70	8.19	24.17	26.26	26.25	24.68

**Table 8:** Performance comparison of cement pastes admixed with ETRI and a set retarder.

Admixture	Dosage	Heat Flow		28 d Compressive Strength (MPa)
		Peak Heat Release Rate (mW/g)	Reduction Rate (%)	
Blank	0	3.11	/	41.13
ETRI	1.50%	0.81	74.12%	48.03
Sucrose	0.07%	2.22	28.82%	43.07
Sodium gluconate	0.07%	2.43	21.87%	44.77
Citric acid	0.07%	2.21	28.94%	43.83

## Data Availability

The original contributions presented in this study are included in the article. Further inquiries can be directed to the corresponding author.

## Abbreviations

The following abbreviations are used in this manuscript:

ETRI	Ester-based polymeric hydration temperature rise inhibitor
C_2_S	Dicalcium silicate
C_3_S	Tricalcium silicate
C_3_A	Tricalcium aluminate
XRD	X-ray diffraction
TGA	Thermogravimetric analysis
ICP-OES	Inductively coupled plasma optical emission spectrometry
MIP	Mercury intrusion porosimetry
SEM	Scanning electron microscope
MA	Maleic acid
AR	Analytical reagent
EG	Ethylene glycol
HQ	Hydroquinone
PTS	P-toluenesulfonic acid
FTIR	Fourier transform infrared spectroscopy
GPC	Gel permeation chromatography
CH	Calcium hydroxide
C-(A)-S-H	Calcium (alumino)silicate hydrate gel
AFt	Ettringite
